# Less Severe Cases of COVID-19 in Sub-Saharan Africa: Could Co-infection or a Recent History of *Plasmodium falciparum* Infection Be Protective?

**DOI:** 10.3389/fimmu.2021.565625

**Published:** 2021-02-18

**Authors:** Allan Kalungi, Eugene Kinyanda, Dickens Howard Akena, Pontiano Kaleebu, Innocent M. Bisangwa

**Affiliations:** ^1^Mental Health Section of MRC/UVRI & LSHTM Uganda Research Unit, Entebbe, Uganda; ^2^Department of Psychiatry, College of Health Sciences, Makerere University, Kampala, Uganda; ^3^Department of Psychiatry, Faculty of Medicine & Health Sciences, Stellenbosch University, Cape Town, South Africa; ^4^MRC/UVRI & LSHTM Uganda Research Unit, Entebbe, Uganda; ^5^ATCG Solutions (Uganda) Limited, Uganda Industrial Research Institute, Kampala, Uganda

**Keywords:** SARS–CoV-2, COVID-19, *Plasmodium falciparum* infection, co-infection, Sub-Saharan Africa

## Abstract

Sub-Saharan Africa has generally experienced few cases and deaths of coronavirus disease 2019 (COVID-19). In addition to other potential explanations for the few cases and deaths of COVID-19 such as the population socio-demographics, early lockdown measures and the possibility of under reporting, we hypothesize in this mini review that individuals with a recent history of malaria infection may be protected against infection or severe form of COVID-19. Given that both the severe acute respiratory syndrome coronavirus 2 (SARS-CoV-2) and *Plasmodium falciparum* (*P. falciparum*) merozoites bind to the cluster of differentiation 147 (CD147) immunoglobulin, we hypothesize that the immunological memory against *P. falciparum* merozoites primes SARS-CoV-2 infected cells for early phagocytosis, hence protecting individuals with a recent *P. falciparum* infection against COVID-19 infection or severity. This mini review therefore discusses the potential biological link between *P. falciparum* infection and COVID-19 infection or severity and further highlights the importance of CD147 immunoglobulin as an entry point for both SARS-CoV-2 and *P. falciparum* into host cells.

## Introduction

The corona virus disease 2019 (COVID-19) pandemic continues to spread and ravage the world with more than 79,673,754 confirmed cases including 1,761,381 confirmed deaths as of 28th December, 2020 (1). COVID-19 is caused by a novel coronavirus known as the severe acute respiratory syndrome coronavirus 2 (SARS-CoV-2). Coronaviruses belong to a family of enveloped single-stranded +sense RNA viruses, ranging from 16 to 32 kb in length and can be divided into four major genera (2). The novel SARS-CoV-2 was named so after being reported to be phylogenetically close to the coronavirus that cause severe acute respiratory syndrome (SARS-CoV) (3).

Several studies have been undertaken in search for an effective treatment for COVID-19. A recent review and meta-analysis of 7 randomized clinical trials and 14 cohort studies (20,979 patients) has reported a lack of efficacy for hydroxychloroquine in reducing short-term mortality in hospitalized COVID-19 patients or in reducing the risk of hospitalization in COVID-19 outpatients (4). Randomized controlled trials have recently reported effective treatment against COVID-19. Dexamethasone has been reported to reduce mortality among COVID-19 patients on respiratory support (5) while remdesivir has been reported to reduce both mortality and recovery time among hospitalized adults with COVID-19 pneumonia (6). A total of seven vaccines have also received emergency use authorization/approval to-date in some countries (7) while many more vaccines remain in development. Among these, BNT162b2 (also known as the Pfizer–BioNTech COVID-19 vaccine) has been authorized for emergency use in the United Kingdom, mRNA-1273 (also known as the Moderna COVID-9 vaccine) received emergency use authorization in the United States and Canada, CoronaVac has received emergency authorization for use in China, Sputnik V has been authorized for use among individuals aged 60 years and above in Russia, BBIBP-CorV has received authorization for use in Bahrain, China and the United Arab Emirates while EpiVacCorona is set to be rolled out in Russia. The Oxford-AstraZeneca coronavirus vaccine is the latest to receive approval for use in the United Kingdom as of 30th December, 2020. However, efficacy and long term safety of these vaccines needs to be carefully monitored.

Globally, Africa stands out as the region least affected by the COVID-19 epidemic, with 63,344 reported deaths and 2,239,023 recoveries out of the 2,677,672 reported cases as of 29th December, 2020 (8). The Central, Western and Eastern parts of Africa harbor the greatest burden of malaria as compared with the Northern and Southern parts of Africa. Interestingly, within Africa, the Central, Western and Eastern parts of Africa further stand out to be least affected regions (1,463 reported deaths and 67,026 recoveries out of 73,317 reported cases; 3,187 reported deaths and 214,701 recoveries out of 239,216 reported cases; and 5,917 reported deaths and 250,296 recoveries out of 319,156 reported cases, respectively), as compared to the Northern (23,766 reported deaths and 757,828 recoveries out of 911,486 reported cases) and Southern (29,011 reported deaths and 949,172 recoveries out of 1,134,497 reported cases) parts of Africa as of 29th December, 2020 (8). Although factors such as extent of testing and patient demographics may be responsible for the observed regional differences in the effect of the COVID-19 pandemic, the Central, Western and Eastern parts of Africa conspicuously stand out as regions most affected by malaria globally. However, it is worthy of note that the trajectory of COVID-19 pandemic in sub-Saharan Africa is not well-known due to a paucity of seroprevalence data. A study by Uyoga et al. (9) has reported a SARS-CoV-2 seroprevalence of 5.63 (4.83–6.49) among Kenyan blood donors while a SARS-CoV-2 seroprevalence of 12.3 (9.0–15.7) has been reported among health care workers in Malawi (10). These seroprevalences are higher than the global pooled seroprevalence of 3.38 (3.05–3.72) that has been reported by a systematic review and meta-analysis (11), suggesting that there are possibly many more infections that are possibly asymptomatic or mild in nature.

Africa is the continent with the highest burden of malaria globally accounting for 93% of global malaria cases and 94% of malaria deaths (12). It is worthy of note that more than half of global malaria cases in 2018 were attributed to 6 African countries, that is Nigeria (25%), the Democratic Republic of the Congo (12%), Uganda (5%), and Côte d'Ivoire, Mozambique and Niger (4% each) (12). We hypothesize that the low effects of COVID-19 observed mainly in sub-Saharan Africa is due to interplay between active or a recent history of *Plasmodium falciparum (P. falciparum)* infection with COVID-19 disease acquisition or progression. We discuss below the association between *P. falciparum* infection and COVID-19 infection or severity. We further discuss the importance of CD147 as an entry route for both SARS-CoV-2 and *P. falciparum* into host cells.

## Discussion

### *Plasmodium falciparum* and SARS-CoV-2 Co-infection

As of 29th December, 2020, With 2,677,672 confirmed cases and 63,344 confirmed deaths (8), Africa remains the least hit continent by the COVID-19 pandemic sharing only around 3.4 and 3.6% confirmed cases and deaths, respectively, of the world's 79,673,754 confirmed cases and 1,761,381 confirmed deaths (1) as of 29th December, 2020. Increasing malaria incident rates have been reported to be associated with decreased COVID-19 incident rates (13, 14). It has been hypothesized that induction of interferons and neutralizing antibodies due to chronic infection by *P. falciparum* may contribute to natural immunity against SARS-CoV-2 in *P. falciparum* endemic areas (15).

Chloroquine/hydroxychloroquine pre-exposure prophylaxis has been suggested as the reason behind the low incidences of COVID-19 in malaria endemic countries (16). However, it should be noted that chloroquine/hydroxychloroquine are no longer first line drugs for treatment of *P. falciparum* malaria and the World Health Organization recommends artemisinin-based combination therapies for the treatment of uncomplicated *P. falciparum* malaria (17) highlighting that other factors such as population socio-demographics, immunological factors and genetic susceptibility could be at play. For example, previous exposure of children to coronavirus OC43 could be protective against severe COVID-19 through cross-immunity since SARS-CoV-2 is closely related to coronavirus OC43 (18). An epidemiological study in a *P. falciparum* endemic area in India has also reported a negative correlation between 10-year annual parasite index scores and the number of COVID-19 cases (19).

The immunoglobulin CD147 has been reported to form a complex with CD98 (CD147-CD98 protein complex) that is involved in the attachment and entry of viruses like Chikungunya into human cells (20), highlighting its importance in viral infection of host cells. Elevated plasma levels of CD147 have been reported among patients with diabetes as compared to healthy controls and predicted mortality over a 10-year period among the diabetic patients (21). The elevated plasma levels of CD147 among patients with diabetes may be due to overexpression of CD147 as a transmembrane glycoprotein. Overexpression of CD147 on host cells may facilitate more viral entry into host cells and may partly explain the high mortality of COVID-19 that has been reported among patients with diabetes. The CD147 has been reported to facilitate entry of SARS-CoV-2 and *P. falciparum* into host cells (22, 23). We therefore hypothesize that the immunological memory elicited following binding of *P. falciparum* to CD147 is protective against SARS-CoV-2 infection or severe COVID-19. We highlight below the probable mechanisms through which *P. falciparum* infection may be protective against SARS-CoV-2 infection or severe COVID-19. We further highlight the importance of CD147 as an entry point for SARS-CoV-2 into host cells.

Glucose-6-phosphate dehydrogenase (G6PD) has also been suggested to play a role in COVID-19 infection and severity (24) and has also been associated with infection of cells with human coronaviruses (25). However, inherited deficiency of erythrocytes G6PD has been reported to confer protection against severe *P. falciparum* malaria (26). It has also been hypothesized that protection against severe malaria is through early phagocytosis of G6PD-deficient erythrocytes that are parasitized by *P. falciparum*. We hypothesize that a recent *P. falciparum* infection may offer the host an immunological memory that marks a SARS-CoV-2-CD147 infected cell for early phagocytosis. Interestingly, reports from the United Kingdom and the United States indicate increased rates of COVID-19 infection and mortality among Blacks and Asians. The increased vulnerability to COVID-19 infection among Blacks in the United Kingdom and the United States may thus be attributed to inherited G6PD deficiency and subsequent lack of the protective effect of *P. falciparum* infection history. However, the increased vulnerability could be due to socio-economic conditions, inability to be socially distanced and higher rates of hypertension and obesity that have been reported most especially among Blacks in the United States.

A study by Iesa et al. (27) has conducted a search of B- and T-cell immunodominant epitope as well as B- and T-cell major histocompatibility complex (MHC) restricted epitopes for shared sequences between *P. falciparum* and SARS-CoV-2. They reported no significantly shared homology between *P. falciparum* B-cell epitopes with SARS-CoV-2, suggesting that no antibodies to *P. falciparum* could be proposed as eliciting an immune response against infection with SARS-CoV-2 through cross-reactivity. However, Iesa et al. (27) observed more than 40% of identities between SARS-CoV-2 N-protein (amino acids 215–227) and *P. falciparum* thrombospondin-related anonymous protein (TRAP) epitopes located at amino acids 509–523 and also between SARS-CoV-2 open reading frame 1ab (ORF1ab) and TRAP (amino acids 101–130). The immunological memory elicited following *P. falciparum* infection could be protective against SARS-CoV-2 infection or severe COVID-19 through the original antigenic sin theory (28), where a second antigen relies on the memory established by the first antigen to initiate response. Iesa et al.'s study has revealed several conserved tetrapeptides and pentapeptides between *P. falciparum* and SARS-CoV-2 in the T-cell immunodominant epitope and T-cell MHC restricted epitopes. Both epitopes have been reported to stimulate CD8^+^ T-lymphocyte response through the HLA-A.02:01 recognition and it has been suggested that the memory of the cellular adaptive immunity mounted against the TRAP-immunodominant epitope could recognize the 219-LALLLLDRL-227–HLA-A^*^02:01 complexes originating from SARS-CoV-2 infection in malaria-endemic regions and trigger an immune response (27). Much as these hypotheses need to be tested, we acknowledge that the protective effect of *P. falciparum* against COVID-19 infection/severity may be due to other biochemical or immunological processes that need to be elucidated.

It is also worthy of note that many areas of Africa such as Rwanda, Central and North Eastern Kenya and vast areas of Ethiopia have quite low malaria incidences, yet they do not seem to be more highly impacted by COVID-19. If *P. falciparum* infection is indeed protective against SARS-CoV-2 infection or severe form of COVID-19, protection in such areas with low *P. falciparum* infection could be due to a long lived immunological memory. For example, antibodies against *P. falciparum* have been reported to persist with a half-life of 5–10 years (29, 30).

### Role of CD147 Receptor in *Plasmodium falciparum* and SARS-CoV-2 Infection

The primary route of infection by SARS-CoV-2 is facilitated by its ability to attach to host cell receptor where they (SARS-CoV-2) enter host cells through various mechanisms. SARS-CoV-2 has been reported to infect host cells through interaction of its spike (S) protein with angiotensin-converting enzyme 2 (ACE2), in a process that is primed by the trans-membrane serine protease 2 (31–35). Entry through endocytosis has also been described facilitated by the S-protein, whose S1 sub unit facilitates receptor binding while the S2 subunit facilitates membrane fusion (36). Endocytosis occurs through clathrin-coated pits, where there is abundance of phosphatidylinositol binding clathrin assembly protein (PICALM) (37).

The CD147 (also known as basigin) has also been reported as an entry route for SARS-CoV-2 into host cells (22, 38, 39) although a study by Shilts and Wright (40) has reported no evidence for CD147 as a direct SARS-CoV-2 spike binding receptor. The CD147 belongs to the immunoglobulin superfamily and has been reported to be a highly glycosylated trans-membrane protein (41, 42). The CD147 protein is present in many cell types, including leukocytes, endothelial haematopoietic and epithelial, endothelial cells where they are expressed at varying expression levels (43, 44). The CD147 has been reported to support platelet–monocyte interactions and also to promote monocyte recruitment to the arterial wall (45). The CD147 has been described to present as a possible novel target to counter vascular inflammation and atherosclerotic lesion development (45).

The CD147 receptor is among a plethora of molecules that play essential roles in invasion of erythrocytes by *P. falciparum* (46). Both CD147 and Cyclophilin B have been reported to bind to merozoites simultaneously, thereby indicating that these proteins bind to independent ligands on the merozoite surface that are, most likely, *P. falciparum* Reticulocyte Binding Protein Homolog 5 (PfRh5) and *P. falciparum* rhoptry-associated protein complex 3 (PfRhopH3) respectively, (47). Inhibition of CD147 through anti-CD147 antibodies has also been reported to block parasite invasion (23). Crosnier and colleagues reported that MEM-M6/6 antibody almost completely blocked the invasion of the parasite at the concentration of 10 μg/ml (23).

## Conclusion

There is enough evidence for both low infection rates and less severe COVID-19 disease in *P. falciparum* endemic areas. The biology underlying this observation needs to be elucidated in order to understand the pathophysiology of COVID-19 disease. The CD147 may as well-need attention as an entry route for both SARS-CoV-2 and *P. falciparum* into host cells.

## Future Steps

There is need for studies to investigate the role of *P. falciparum* infection on the etiology of COVID-19. Studies that compare outcomes in patients with a recent history of *P. falciparum* infection with those in patients with no recent *P. falciparum* infection coupled with studies that provide immune profiles in patients infected with *P. falciparum*, SARS-CoV-2, and *P. falciparum*-SARS-CoV-2 co-infection may provide a stepping stone. More experimental studies are also required to investigate the potential role of *P. falciparum* infection on SARS-CoV-2 infection or COVID-19 severity.

## Author Contributions

AK and IMB provided concept. AK did literature review and first draft. AK, EK, PK, DHA, and IMB did final revision. All authors read and approved the final manuscript.

## Conflict of Interest

IMB is a director at ATCG Solutions (Uganda) Limited, a molecular diagnostics laboratory that had no commercial interest in the present study. The remaining authors declare that the research was conducted in the absence of any commercial or financial relationships that could be construed as a potential conflict of interest.

## Abbreviations

CD: cluster of differentiation
COVID-19: coronavirus disease 2019
G6PD: glucose-6-phosphate dehydrogenase
PICALM: phosphatidylinositol binding clathrin assembly protein
SARS-CoV-2: Novel coronavirus.
